# American Academy of Dental Science

**Published:** 1900-12

**Authors:** 

**Affiliations:** American Academy of Dental Science


### Reports of Society Meetings.

### AMERICAN ACADEMY OF DENTAL SCIENCE.

THE regular monthly meeting of the American Academy of Dental Science was held at Young's Hotel, Wednesday evening, October 3, 1900, at six o'clock.

A paper was read by William H. Potter, D.M.D., of Boston, entitled "Some German Literature of Interest to the Profession."

(For Dr. Potter's paper, see page 781.)

*Dr. Potter.*—I am put down on the card to show a dissection of the lower maxilla, and I now present it. I had it made for the Harvard Dental School, in order to be able to show the relation between the contents of the inferior dental canal and the roots of the teeth. You will also notice a dissection of the mental nerve and artery.

There have been one or two cases reported of late where, in lancing an abscess at the end of a lower bicuspid, the mental nerve has been severed; and it seemed to me quite important to have a dissection so that we could impress upon the minds of students the conditions to be met in this region.

It seems to me that the next great advance in dental science will come from the work of the laboratory, and that therefore the scientific side of our profession is of the greatest importance. The treatment of affections of the tooth socket is, in my opinion, the greatest problem which is before us. A treatment based on pure mechanics does not suffice. The problem must be dealt with in the laboratory, by a well-equipped scientific investigator. There are men abroad in the practice of dentistry who seem to me better fitted by education to undertake such work than are we.

### DISCUSSION.

*President Pond.*—The subject is open for discussion.

*Dr. Werner.*—I envy Dr. Potter for the opportunities, and for the kindly reception that I know he would meet wherever he went in Europe. I think some men over there envied him for his opportunities for what they think we in America have. Of course, I am enough of a German, and perhaps know enough of Germany, to know that they have some very scientific men there. Their best talent there is the best talent; at least, there is none better. I think, as he does, that it is the men in the laboratories, the men who have that broad education in general medicine, who are going to be the means of advancing our specialty in years to come, further than any other class of men.

And yet I am far from one who underestimates the mechanical, the technique, in dentistry. The mechanical side seems so thoroughly and comprehensively developed that the further advance must come in conjunction with bacteriology and physiological chemistry.

Generally speaking, we have a very vague idea of how dentistry is taught in European schools. My little investigations on my trips there have opened my eyes. There you cannot find a dental school as we know dental schools to have existed in America, —namely, exclusively dental schools. It would seem to them ridiculous in a dental department to teach only the anatomy of the head and neck, and to stop there; it would seem to them the essence of superficialness. Most of our dental schools in America were instituted with very limited medical instruction. Gradually they have developed a broader curriculum, so as to give a thorough training in anatomy, physiology, and chemistry.

In Austria and Germany, and practically in France and Italy and England, the "Zahnarzt," or dental physician, is a person looked up to. He cannot have the title, and cannot put on his sign "Zahnarzt," without his qualification back of it; he would be prosecuted at once, and not by a political board of examiners, as with us in America, but in an entirely different way. While I do not wish to speak slightingly of our State boards of dental examiners, I do think they are wrongly constituted, have too much power, are too much of a political force. They should be differently appointed, and they should have to undergo an examination. The Germans have it nearer. Once examined, not by a political board, but by a board constituted of competent teachers, he is licensed to practise, not in one state, but in the whole empire. Our present board of examiners should be gradually modified in that direction.

Then I know how thoroughly up to date the Germans are in everything pertaining to bacteriology, physiology, and chemistry. In order to bring our specialty to a higher point, we will need to improve our dental education in bacteriology and physiological chemistry.

I wish Dr. Potter had spoken of many more points that would have been of interest; for I think visits like his, finding out what somebody else has and what he does not have, what he is taught and what he is not taught, is of great benefit to us. It is also a good thing for you and me to see some one else operate, to see his office, to see him do his work. We can learn something from most any one, and perhaps help him to improve in certain directions.

*Dr. Baker.*—Mr. President, will Dr. Potter explain how this specimen was preserved?

*Dr. Potter.*—It was preserved in a formalin solution while being dissected; it was sent from Vienna to Boston in a sealed zinc box. There was a small amount of formalin in the box, about half a litre, and the extra room was filled with excelsior. In Boston the specimen was transferred to alcohol. Alcohol hardens a specimen more than does a formalin solution.

In Vienna they use formalin almost exclusively for anatomical specimens. They have great tanks of it, and bring out from them for the day's lecture the required amount of anatomical material, and then put it back to remain for perhaps a year.

*Dr. Stevens.*—What is the significance of the fringed tongue? Does it signify any disease?

*Dr. Potter.*—I do not know just what Dr. Stevens means by the fringed tongue. The affection which I have called attention to under the name of "lingua geographica" has no serious signification.

*Dr. Stevens.*—I thought it had an important signification.

*Dr. Potter.*—Not what I called lingua geographica.

*Dr. Stevens.*—It is not syphilis?

*Dr. Potter.*—The book which I have quoted, "Die Krankheiten des Mundes, von J. Mikulicz und W. Kümmel," says that two writers relate it to syphilis, but that almost all writers say that it has nothing to do with that affection.

*Dr. Stevens.*—That is what I supposed.

*Dr. Potter.*—As I remember it, it is that way. Some have thought it was syphilitic, but he considers it rather more interesting than serious.

*Dr. Smith.*—Does he outline any treatment?

*Dr. Potter.*—The author quoted says that in such cases treatment is unnecessary.

*Dr. Werner.*—As I understand it, it is not "fringed tongue," but the geographical or map-like divisions the papillæ make, the little grooves that divide them into sections and pictures. I have a family where such a tongue is very distinct, and where there are no signs of syphilitic lesions in the mouth. I have seen it described in German books.

*Dr. Fillebrown.*—I wish to thank Dr. Potter for what he has done for the Academy and for the profession. I was familiar with the doctor's intention when he went abroad, and took a great interest in his doings while away, and the results which he has brought home justify my expectations. The literature he has brought and his interpretation of its bearing upon our wants are interesting and valuable. The problem of harmonizing and unifying science and practice is really with us a difficult one. Ours is the profession of applied science. The problem of our profession to-day is the problem of applying science. Dentistry began at the practical end of the subject, and I think there is no question but what that end is well mastered, and, as has been remarked here, our principal improvement is to be in following the paths as they are further marked out by scientific study and scientific application in the laboratory.

I remember some years ago I considered the subject of the "Influence of Culture on Professional Skill." I took the ground then, and I take the ground now, that whatever you do must first be conceived in the mind. You cannot make a figure, you cannot form a letter, or even make a straight line, until the conception is formed in the mind. There is also no question but what the doing reacts upon the mind and aids in perfecting the conception.

And so in the field of dentistry, the farther we go in our scientific applications, the better we can understand our anatomy, our physiology, chemistry, and bacteriology, the truer will be our mental conception, and the better we can carry it out with the hand.

Subject passed.

*President Pond.*—The next in order of business is "Incidents of practice and presentation of specimens." Has any gentleman anything to offer?

*Dr. Potter.*—I do not think I can resist showing you a good syringe. I have always considered the ordinary syringe with leather packing as unfit to use; it is an unclean instrument. Nowadays people are beginning to open their eyes in regard to syringes, and are making syringes which are fit to use in the mouth.

I looked over the syringes for sale in Berlin and Vienna, and found the one which I have here to-night. The piston is entirely made of metal; it is accurately ground to the barrel, and is a satisfactory plunger. Before using the syringe the piston should be touched with vaseline or glycerin. I prefer glycerin because it washes off more easily than vaseline. This syringe can be boiled in all its parts without the slightest injury.

*Dr. Baker.*—There is no packing in the instrument?

*Dr. Potter.*—Not the slightest. I wish you would test the instrument. I have used it for several months, and therefore have more confidence in speaking of it.

*Dr. Williams.*—What is the metal?

*Dr. Potter.*—The barrel is brass and the plunger nickel.

*Dr. Werner.*—How often do you boil it?

*Dr. Potter.*—Every time I use it.

On motion, adjourned.

Charles H. Taft, D.M.D.,
*Editor American Academy of Dental Science.*

